# Closed-loop control of k-space sampling via physiologic feedback for cine MRI

**DOI:** 10.1371/journal.pone.0244286

**Published:** 2020-12-29

**Authors:** Francisco Contijoch, Yuchi Han, Srikant Kamesh Iyer, Peter Kellman, Gene Gualtieri, Mark A. Elliott, Sebastian Berisha, Joseph H. Gorman, Robert C. Gorman, James J. Pilla, Walter R. T. Witschey

**Affiliations:** 1 Department of Bioengineering, Jacobs School of Engineering, University of California, San Diego, CA, United States of America; 2 Department of Radiology, School of Medicine, University of California, San Diego, CA, United States of America; 3 Department of Medicine, Perelman School of Medicine, University of Pennsylvania, Philadelphia, PA, United States of America; 4 Department of Radiology, Perelman School of Medicine, University of Pennsylvania, Philadelphia, PA, United States of America; 5 National Heart Lung and Blood Institute, National Institutes of Health, Bethesda, MD, United States of America; 6 AJO, Philadelphia, PA, United States of America; 7 Department of Surgery, Perelman School of Medicine, University of Pennsylvania, Philadelphia, PA, United States of America; Universitatsklinikum Wurzburg, GERMANY

## Abstract

**Background:**

Segmented cine cardiac MRI combines data from multiple heartbeats to achieve high spatiotemporal resolution cardiac images, yet predefined k-space segmentation trajectories can lead to suboptimal k-space sampling. In this work, we developed and evaluated an autonomous and closed-loop control system for radial k-space sampling (ARKS) to increase sampling uniformity.

**Methods:**

The closed-loop system autonomously selects radial k-space sampling trajectory during live segmented cine MRI and attempts to optimize angular sampling uniformity by selecting views in regions of k-space that were not previously well-sampled. Sampling uniformity and the ability to detect cardiac phase in vivo was assessed using ECG data acquired from 10 normal subjects in an MRI scanner. The approach was then implemented with a fast gradient echo sequence on a whole-body clinical MRI scanner and imaging was performed in 4 healthy volunteers. The closed-loop k-space trajectory was compared to random, uniformly distributed and golden angle view trajectories via measurement of k-space uniformity and the point spread function. Lastly, an arrhythmic dataset was used to evaluate a potential application of the approach.

**Results:**

The autonomous trajectory increased k-space sampling uniformity by 15±7%, main lobe point spread function (PSF) signal intensity by 6±4%, and reduced ringing relative to golden angle sampling. When implemented, the autonomous pulse sequence prescribed radial view angles faster than the scan TR (0.98 ± 0.01 ms, maximum = 1.38 ms) and increased k-space sampling mean uniformity by 10±11%, decreased uniformity variability by 44±12%, and increased PSF signal ratio by 6±6% relative to golden angle sampling.

**Conclusion:**

The closed-loop approach enables near-uniform radial sampling in a segmented acquisition approach which was higher than predetermined golden-angle radial sampling. This can be utilized to increase the sampling or decrease the temporal footprint of an acquisition and the closed-loop framework has the potential to be applied to patients with complex heart rhythms.

## Introduction

Cine MRI captures the motion of the heart by acquiring images at frame rates faster than the motion occurs. One approach to do so is to collect the image at a frame rate much higher than the heart rate [1, 2], yet practical limitations of physiology, hardware and patient safety limit how quickly image data can be obtained and spatial or temporal fidelity may be compromised. The lowest acceptable frame rate to visualize a heart beating at 60 beats-per-minute is about 20 frames-per-second and higher frame rates are required for higher heart rates [3]. To improve fidelity, cine MRI is conventionally performed using segmented sampling techniques where periodic motion enables a subset of k-space data to be collected [4–6].

Segmented MRI can be performed using different k-space trajectories including Cartesian and non-Cartesian patterns. However, Cartesian trajectories are known to be sensitive to arrhythmias and data from irregular beats must be reacquired otherwise images will have inconsistent spatial information and artifacts. While arrhythmia rejection algorithms have been developed, reacquiring rejected data can lead to prolonged breathholds that are too long for patients–which leads to respiratory motion artifacts. Non-Cartesian radial [7–10] and spiral trajectories [11, 12] have the advantageous properties of local k-space uniformity, meaning that adjacent lines of data can be distributed uniformly in k-space, reducing the need for prolonged acquisitions. In particular, golden angle acquisitions [13] can address the problem of arrhythmias by using an adaptive temporal footprint [14, 15]. However, a limitation of this approach is that only a contiguous set of golden angle views will have uniform k-space sampling properties. As a result, segmented golden angle trajectories are suboptimal, since views collected from adjacent heartbeats will not have near uniform k-space sampling [14, 16, 17].

To address this limitation, we developed a radial trajectory that adapts in response to physiologic changes in the patient being scanned and uses same-scan data to optimize the sampling trajectory on-the-fly using a closed-loop [18]. In this manuscript, we evaluate the sampling uniformity and point spread function signal properties using ECG data from subjects with normal rhythm. Further, we demonstrate the autonomous radial k-space sampling (ARKS) control system on a whole-body clinical MRI scanner. We show feasibility of this approach in 4 healthy human volunteers and demonstrate initial proof of utility in one patient with a complex rhythm. Images and sampling properties were compared to a segmented golden angle cine MRI approach.

## Methods

In this study, all subjects and patients gave written informed consent prior to participating in the study, approved by the Institutional Review Board of the University of Pennsylvania.

### Closed-loop radial k-space sampling

We developed a closed-loop radial acquisition in which the k-space trajectory to be acquired is calculated dynamically throughout the scan. The data to acquire is determined according to the segmented data which was previously acquired with the goal of minimizing angular gaps in k-space and reducing unequal angular sampling density. All acquired k-space locations are time-stamped and a simple cross-correlation of the ECG signal is used to identify prior periods of similar cardiac phase. The advantage of ECG-matching by cross-correlation is that it has real-time performance and can compute cross-correlation results in less than 1 ms—an essential requirement for fast scans such as segmented cine MRI which requires very rapid repetition times.

**Fig 1:**
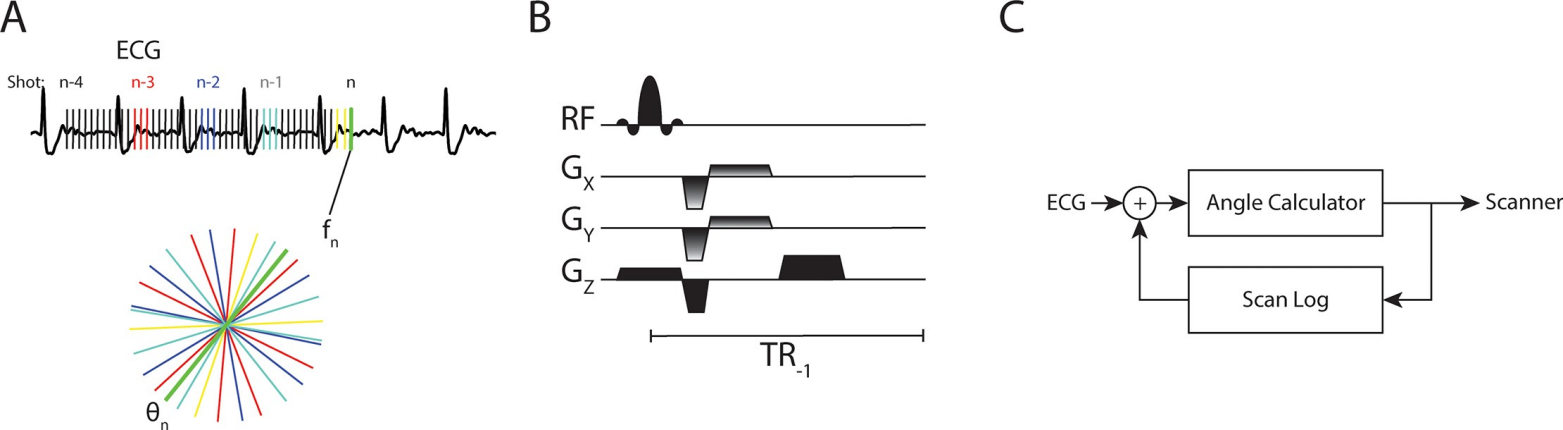
Segmented acquisition using an autonomous closed-loop system. **A)** During the acquisition, the closed-loop system identifies prior, similar phases of the cardiac cycle using cross-correlation of the recorded ECG signal. For frame f_n_, radial k-space lines from prior phases (red, blue, and teal) as well as the most recent views (yellow) are aggregated and the newest projection θ_n_ (green) is defined to bisect the largest angle, thus closing the gap in k-space. **B)** The calculated angle θ_n_ is provided to the bSSFP sequence in real-time such that the Gx and Gy gradients are updated. **C)** The closed loop nature of the physiologic signal (ECG), the angle calculator, scanner, and log of prior scan data.

An overview of the closed-loop radial k-space sampling acquisition is shown in **Fig 1** and more details are included in **S1 Fig**. Prior periods of similar ECG signal are identified as local maxima of the cross-correlation of the historical signal with the most recent ECG signal. In conventional segmented k-space trajectories, the total number of projections *N*_*θ*_ is obtained by sampling a subset of radial views (segments *N*_*s*_) during each heartbeat (shots *N*_*q*_). **Fig 1** shows how segments and shots are defined for a closed-loop acquisition. In our closed-loop approach the definition of *N*_*θ*_ is different from a traditional segmented trajectory, for which *N*_*θ*_ =*N*_*S*_*N*_*Q*_, as closed-loop sampling can only use half the views of the current shot since the other half has not occurred yet. For the four heartbeat segmented cine example shown in **Fig 1** there are 3 shots with 4 segments (i.e. radial views), but the fourth shot (yellow) has only 2 views.

From the list of all previously acquired view angles, the subset corresponding to the periods associated with local maxima in the cross-correlated ECG signal are collated. The new radial angle *θ*_*i*_ (green radial line in **Fig 1A**) is chosen so that it bisects the largest angular gap Δ*θ*_*i*_. As a result, the closed-loop acquisition closes the largest gap in k-space and improves angular sampling uniformity.

### Implementation and interface of closed-loop radial scheme with MRI system

To demonstrate the feasibility of autonomous radial imaging, we developed a software platform for closed-loop radial imaging and interfaced it to a whole-body clinical MRI scanner. As outlined in **S2 Fig**, the system consisted of four systems for real-time communication and feedback: (1) the physiologic monitor, (2) adaptive measurement controller, (3) pulse sequence, and (4) digital signal processors (DSPs).

ECG signals were received from the patient using a MR-compatible 4-lead system (In vivo Gainesville, FL USA). The ECG signal was transmitted wirelessly to the physiologic monitor. Analog signals were digitized, logged and transmitted via TCP/IP in software (LabView, National Instruments, Austin, TX USA) to the adaptive controller, which in turn determined a new view angle.

During the closed-loop period, the adaptive measurement controller and pulse sequence respond to ECG feedback. The adaptive measurement controller performs 3 steps sequentially. It a) transmits an updated k-space trajectory to the pulse sequence; b) reads and stores in memory new ECG data; and c) analyzes ECG data and computes a new k-space trajectory. The pulse sequence receives the new trajectory from the adaptive measurement controller, transmits the instructions to the DSPs and enters the standby period until the DSPs have executed a wake-up instruction. After wake-up, the pulse sequence waits for a new trajectory from the adaptive measurement controller and repeats.

To evaluate real-time timing performance, the calculation times for the adaptive measurement controller were measured for the initialization and active periods of the software. Average, standard deviation, and maximum calculation times were calculated. Timing information was calculated during scanning of 4 subjects. For each subject, 4 scans were performed according to the segmentation strategies in **Table 1**. The average calculating time for the initialization period was 0.793 ± 0.030 ms and for active mode was 0.98 ± 0.01 ms. The maximum time to update was 1.11 ms during the initialization period and 1.38 ms during the active period was. All maximum update times were faster than the repetition time of the sequence.

**Table 1:** Performance of closed-loop radial imaging sampling for 10 subjects with recorded ECG for different k-space segmentation sampling schemes.

N_θ_	N_q_	N_s_	Uniformity (%)^§^			PSF Signal Ratio		
			ARKS	Golden	Random	ARKS	Golden	Random
27	1	54	94.6±1.0*	82.9±0.0	50.9±0.1	99.8±0.2*	94.9±0.0	86.5±0.3
	2	18	71.5±0.6*	66.9±1.5	50.9±0.1	93.3±0.2*	91.2±0.8	86.6±0.2
	5	6	69.0±1.1*	56.5±1.8	50.9±0.1	91.9±0.4*	87.5±0.9	86.6±0.2
45	1	90	94.4±1.1*	83.8±0.0	50.6±0.2	99.3±0.2*	90.3±0.0	78.7±0.2
	2	30	70.4±0.7*	66.1±3.4	50.6±0.1	88.6±0.1*	86.6±1.0	78.8±0.2
	5	10	68.0±1.1*	56.3±3.5	50.5±0.1	86.7±0.7*	81.2±1.6	78.9±0.2
	8	6	66.5±1.3*	54.3±2.4	50.6±0.1	85.8±1.1*	80.3±1.0	78.9±0.2
75	1	150	94.4±0.8*	82.0±0.0	50.3±0.1	98.9±0.5*	87.9±0.0	74.7±0.4
	2	50	69.5±0.6*	64.7±4.3	50.4±0.2	86.3±0.8*	85.6±0.7	74.8±0.3
	8	10	65.8±1.0*	53.5±4.3	50.3±0.1	83.1±1.0*	78.4±1.6	74.8±0.3

^**§**^Uniformity (Eq 12) compares the performance of the radial acquisition to the ideal distribution (Eq 9).

*indicates significant (p<0.05) difference between ARKS and golden approach. N_θ_ = number of radial views, N_q_ = number of shots, N_s_ = number of segments/shot, ARKS = autonomous radial k-space sampling, GA = golden angle, PSF = point spread function.

### Simulation-based assessment of closed-loop k-space trajectory performance

We performed simulations using recorded ECG signals to investigate the distribution of view angles that would be assigned under a closed-loop radial trajectory. This simulation did not include k-space or image space data but was used to evaluate the distribution of view angles for different ECGs with respect to angular sampling density (uniformity) and their point spread function (PSF). For this simulation, 3-lead, chest ECG data was collected from 10 normal subjects at a 400 Hz sampling rate and resampled to the MRI scanner repetition time (TR). The duration of the ECG recordings was 75.7 ± 23.7 sec, corresponding to 2.7 ± 0.85 x10^4^ views at TR = 2.8 ms.

After an initial training period where golden angle radial sampling *θ* = 111.25° occurred, ECG signals were cross-correlated, and view angles were determined for the segmentation schemes shown in **Table 1**. These results were compared to segmented golden angle and random radial approaches. Golden angle views continuously incremented at the golden angle following the initialization period. Random view angles were chosen from the interval 0 to 180° with uniform (flat) probability distribution for selection.

A second set of closed-loop radial simulations were performed using ECG and previously collected cardiac cine data from one patient with an arrhythmia. Image data was acquired on a 1.5 T whole-body MRI system (Avanto; Siemens Healthcare; Erlangen, Germany) equipped with a 40 mT/m gradient coil and a 32 channel RF receiver array (16 anterior and 16 posterior elements). Cardiac gating was obtained with a 3-lead wireless ECG system. Time-stamps were communicated using TCP/IP from the pulse sequence to the ECG log file to synchronize imaging and ECG data. Left ventricular, short-axis, real-time data was obtained using a golden angle radial trajectory and image parameters, flip angle = 70°, TE = 1.4 ms, TR = 2.8 ms, number of frequency encoded points = 128, field-of-view = 340 mm x 340 mm, slice thickness = 8 mm, bandwidth = 1140 Hz/pixel. 16.8 seconds of continuous golden angle radial data was collected, resulting in 6000 golden angle radial projections. K-space signal data was reconstructed offline using adaptive coil synthesis [19] and non-Cartesian SENSE algorithm [20] in open-source software [21] on a Linux workstation as previously detailed [22]. Images were reconstructed with an exposure time (temporal footprint) of 95 ms (= 34 projections per frame) and sliding window of 1 (leading to 357 images per second). To remove residual radial streak artifacts, a median filter was applied with a width of 30 frames. The final 128 x 128 images were interpolated to 512 x 512 to generate the simulation spin density *ρ*(***x***). Simulated k-space data was then generated and sampled using random, golden angle or closed-loop acquisitions. Images were reconstructed as for the in vivo data in the following sections.

### In vivo evaluation of closed-loop MRI imaging and image reconstruction

Imaging was performed on a 1.5 T whole-body MRI scanner (Avanto, Siemens Healthcare, Erlangen, Germany) with a 16-channel RF receiver array. 4 healthy volunteers subjects participated in this study. To visualize cardiac contraction, a mid-ventricular short-axis slice was imaged during an instructed breathhold. Subjects were imaged using a spoiled gradient echo sequence with the following parameters: TE/TR = 4.1/8.2 ms, FOV = 320x320 mm^2^, bandwidth = 240 Hz and flip angle = 12°. The MRI sequence sent a request to the adaptive controller for the next radial angle to acquire 2 ms prior to the end of the current data acquisition (every TR). This 2 ms window allowed for reply from the adaptive controller as well as preparation of the RF gradients by the scanner given the prescribed angle. Acquisitions were performed with various combinations of radial views (segments) per heartbeat (shots) as shown in the **Table 2**.

**Table 2:** Mean and variability of k-space uniformity for golden-angle and autonomous scanning.

N_θ_	N_q_	N_S_	Mean Uniformity		Uniformity Variability		PSF Signal Ratio	
			ARKS	GA	ARKS	GA	ARKS	GA
**27**	5	6	62.7 ± 0.5	56.4 ± 5.9	5.2 ± 0.2	8.0 ± 2.4	87.6 ± 0.7%	84.0 ± 5.6%
**45**	5	10	65.7 ± 2.0	53.0 ± 7.1	4.2 ± 0.1	9.7 ± 3.3	83.2 ± 0.9%	73.2 ± 6.6
**45**	8	6	56.0 ± 1.5	58.3 ± 2.9	4.4 ± 0.2	6.5 ± 1.3	79.4 ± 1.0%	80.2 ± 3.2
**75**	8	10	63.1 ± 1.1	56.4 ± 6.5	3.3 ± 0.2	6.6 ± 2.8	79.6 ± 0.8%	75.4 ± 2.9

N_q_ = number of shots, N_s_ = number of segments/shot, ARKS = autonomous radial k-space sampling, GA = golden angle, PSF = point spread function.

Image reconstruction was performed using a previously-described iterative reconstruction with spatial and temporal variation constraints which also incorporates parallel imaging [23, 24]. As previously described, weights and convergence criteria were empirically tuned on a test dataset to provide high visual quality, while achieving rapid convergence of the cost function and the iterative approach was stopped when the convergence criterion was reached:

$$\frac{100\|I^{n+1}-I^{n}\|}{\left\|I^{n}\right\|}\leq 0.025$$ (1)

### Sampling performance metric: Point spread function analysis

The number of projections *N*_*θ*_ required to fulfill Nyquist sampling and prevent aliasing of the Fourier signal is [25, 26]

$$N_{\theta }=\frac{N_{r}\pi }{2}$$ (2)

where *N*_*r*_ is the number of samples *k*_*r*_ per projection. More generally, the point spread function (PSF) describes degradation of a MR image by the k-space sampling trajectory. In the case of Nyquist sampling, the field-of-view free from image degradation is equal to 1/Δ*k*_*r*_. However, in the case of angular undersampling (*N*_*θ*_≤2*πN*_*r*_), this diameter is reduced by the ratio of the number of projections *N*_*θ*_ to the number of samples per projection *N*_*r*_ [27]

$$FOV=\frac{N_{\theta }}{2\pi N_{r}}\frac{1}{\Delta k_{r}}$$ (3)

In general, Eqs (2) and (3) are correct for a radial sampling distribution in which the view angles are separated by a single angle Δ*θ*. However, while other radial sampling trajectories may not satisfy the same Nyquist sampling criteria given by Eq (2) [27], it is still possible to understand the aliasing properties of the image from the PSF of the sampling trajectory such as for closed-loop sampling. An additional complicating factor is that each image frame does not have the same distribution of Δ*θ* so it is not possible to understand all the aliasing properties of closed-loop radial sampling from a single image frame.

To better understand potential image space artifacts caused by closed-loop radial sampling and segmented golden angle imaging, we estimated the PSF for each image frame individually and combined these individual results to present a single average PSF. We observed that these average results showed circular symmetry on account of the large number of image frames that were involved. The circularly symmetric PSF could thus be presented as a single 1D projection through the center of the 2D PSF. Revolving the 1D circularly symmetric PSF around the coordinate [*k*_*x*_ = 0, *k*_*y*_ = 0] by 180° would recover the original 2D PSF. The PSF signal intensity ratio was estimated from the 1D circularly symmetric PSF as the sum of signal s(x) in the main lobe (-*τ* to *τ* defined by the location of the first zero-corssing) divided by the total signal in the image.

$$PSF\;Ratio=\int_{-\tau }^{\tau }s(x)dx/\int_{-\infty }^{\infty }s(x)dx$$ (4)

### Sampling performance metric: Quantitative analysis of view angle distribution

We analyzed the distribution of view angles for different k-space sampling approaches using probability and cumulative sum functions as illustrated by **S3 Fig.**

The probability distribution *χ* is the probability that two adjacent view angles are separated by Δ*θ* where Δ*θ* lies on the closed interval (0,*π*]. The ideal distribution of *χ*_0_ is the Dirac delta function

$$\chi_{0}=\delta (\Delta \theta_{i}=\frac{\pi }{N})$$ (5)

where all radial view angles are separated by the same angle $\Delta \theta_{i}=\frac{\pi }{N}$ and *N* is the total number of view angles and whose aliasing properties are given by Eq 3.

In general, it should be noted that radial k-space trajectories do not uniformly sample k-space, since the center of k-space has higher sampling density than the periphery. The ideal distribution for radial k-space sampling is only ideal with respect to the Δ*θ* parameter.

The deviation from the ideal distribution can be measured using uniformity metric U which is the ratio of integral of the cumulative sum CS of the sorted vector of view angles relative to the ideal sampling approach where

$$CS(k)=\sum_{i=1}^{k}\Delta \theta_{i}$$ (6)

U=∑k=1NCSχe(k)∑k=1NCSχ0(k) (7)

where ${CS}_{\chi_{e}}$ is the cumulative sum of the trajectory of interest, ${CS}_{\chi_{0}}$ is of the ideal sampling, k lies on the interval (1,N) and N is the number of view differences Δ*θ*. *U* is the uniformity of the sampling distribution and quantitatively compares any view angle distribution to the ideal distribution. Analytical expression for Eqs (6–7) are not available for closed-loop radial imaging since it depends on the ECG, algorithm and acquisition parameters. Uniformity was calculated for each image in the acquisition which enabled both mean and variability (standard deviation) metrics to be defined.

### Statistics

Continuous, quantitative measures such as angular uniformity and PSF ratio are reported as mean values with standard deviations. Given non-normal distribution of the parameters (based on the Shapiro-Wilk test), significant differences in angular uniformity and PSF signal ratio between the proposed closed-loop system and golden angle and random sampling were assessed using the Wilcoxon Signed-Rank test for paired samples at a P < 0.05 level of significance (Matlab, the MathWorks, Natick, MA).

## Results

### Closed-loop, segmented cine MRI acquisition

**Fig 2:**
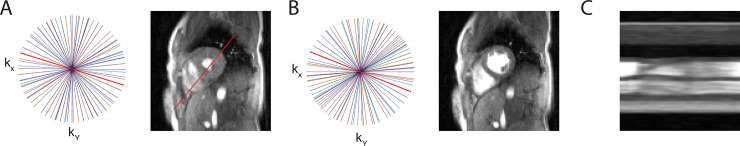
Closed-loop radial sampling of scanning in a healthy volunteer. Distribution of radial views and corresponding 2D real-time short axis images of closed-loop sampling at **A)** end-systole and **B)** end-diastole. Adaptive sampling results in near uniform radial distribution of views and thus high image quality. **C)** Cardiac motion is shown via projection through the left ventricle.

Cardiac cine MRI data was acquired on 4 healthy subjects using the autonomous sampling trajectory at 1.5 T. **Fig 2** shows results from a segmented (eight-shot) acquisition during a breathhold in a normal subject. The adaptive sampling technique showed good sampling uniformity across the cardiac phase. Furthermore, the image quality was good and showed that end-systolic and end-diastolic periods were well-resolved with good ventricular-blood contrast.

Despite being initialized with a golden angle trajectory, the closed-loop approach did not sustain this pattern and within a second the angular gaps deviated substantially from the golden trajectory. Reconstructed, multi-shot images illustrate that ECG cross-correlation can be used to robustly identify the correct cardiac phases. A projection through the left and right ventricle (along the 4-chamber view) is included to demonstrate the motion of the heart (**Fig 2C**). In this view, the motion of the ventricular wall is shown during the scan. In this healthy subject with no history of cardiovascular disease, the wall motion was normal with regular contractile function of the myocardium.

### Point spread function and view distribution analysis

**Fig 3:**
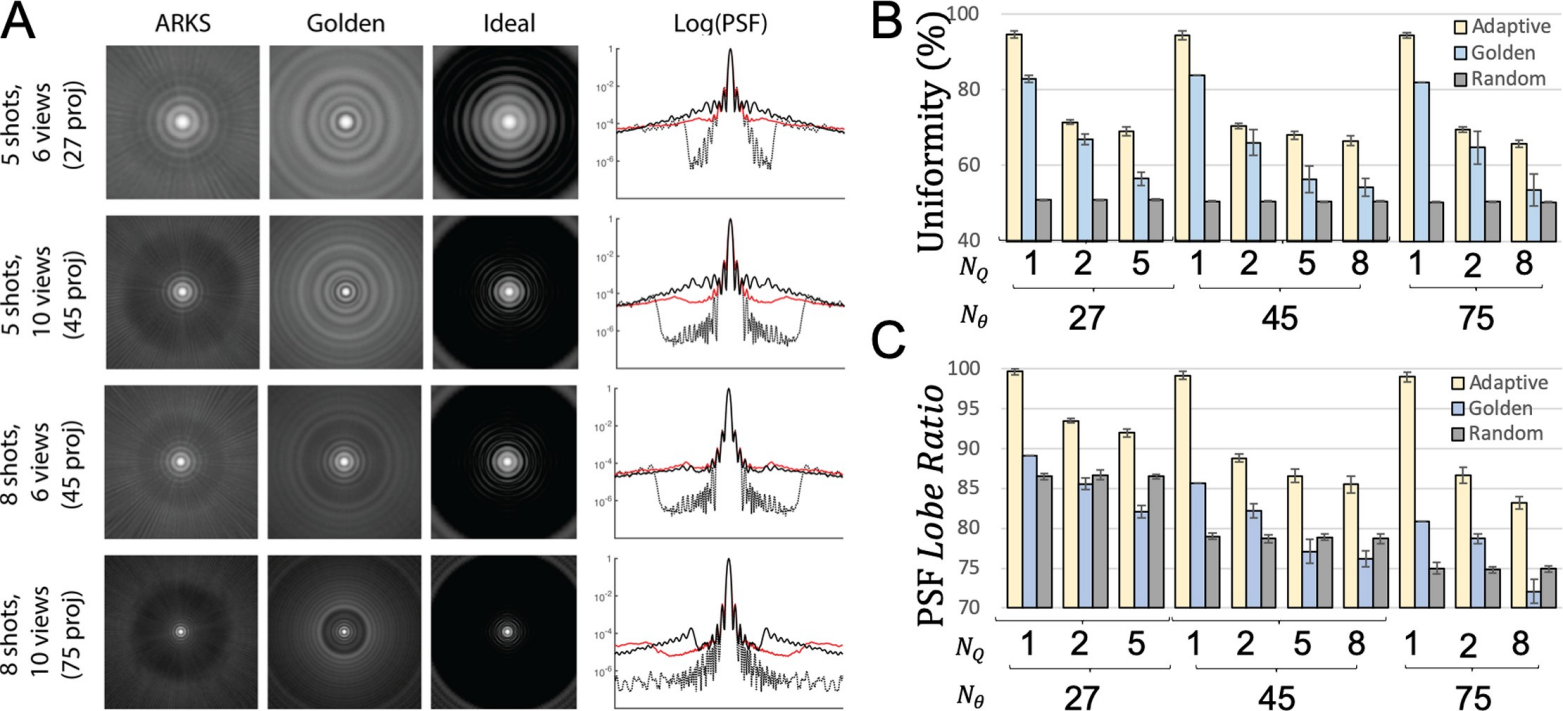
Point spread function and k-space sampling uniformity of autonomous, golden angle, random, and equispaced radial scanning. **A)** Each row on the left shows the 2D point spread function for different segmentation strategies and each column shows autonomous (ARKS), golden or equispaced (Ideal) point spread functions. The fourth column shows a 1D profile for the circularly symmetric PSFs (autonomous shown in red). **B) and C)** K-space uniformity (Eq 7) and the PSF (Eq 4) for ten subjects with different combinations of shots and segments. The autonomous approach results in improved PSF images (left), signal uniformity (top right), and point spread function lobe ratio (bottom right) across all combinations of shots and segments. ARKS = autonomous radial k-space sampling, PSF = point spread function, N_θ_ = number of radial views, N_q_ = number of shots.

During autonomous scanning the closed-loop controller selected new k-space radial projections on-the-fly. Since the user has no control over the trajectory, it was unclear what angles would be chosen and how it would affect image quality, uniformity and the point spread function. **Fig 3** shows that the autonomous scan achieved lower blurring (improved PSF images) than a similar segmented golden angle trajectory.

To obtain a better understanding of these properties, the uniformity of autonomous, golden and random trajectories was compared to the percent ideal uniformity that would be achieved with a constant Δ*θ*. If the trajectory were perfectly uniform, then all views would be equally distributed between 0 and 180°. Per-frame uniformity enabled estimation of uniformity variability (standard deviation of uniformity values over the acquisition).

**Fig 3B** and **Table 1** show that the autonomous scan had the best k-space sampling uniformity for all combinations of segmented trajectories that were investigated for recorded ECGs (**Table 1**). Angular uniformity from autonomous sampling during recorded ECGs was 15±7% (range: 7–23%) higher than golden-angle sampling and PSF signal ratio was 14±6% (range: 1–12%) higher than golden-angle sampling. Single-shot closed-loop trajectories were only slightly lower in uniformity (94.4–94.6) and PSF signal (98.9–99.8) than equispaced sampling where the oldest view would be replaced immediately and surpassed golden angle trajectories in both metrics for all shot/segment combinations.

In the four patients imaged in vivo (**Table 2),** the autonomous approach increased k-space sampling mean uniformity in 3 out of the 4 scan modes (10±11%, range: -4–24%), decreased uniformity variability in all scan modes (44±12%, range: 32–57%), and increased PSF signal ratio in 3 out of 4 scan modes (6±6%, range: -1–14%) relative to golden angle sampling.

### Arrhythmia subject

**Fig 4:**
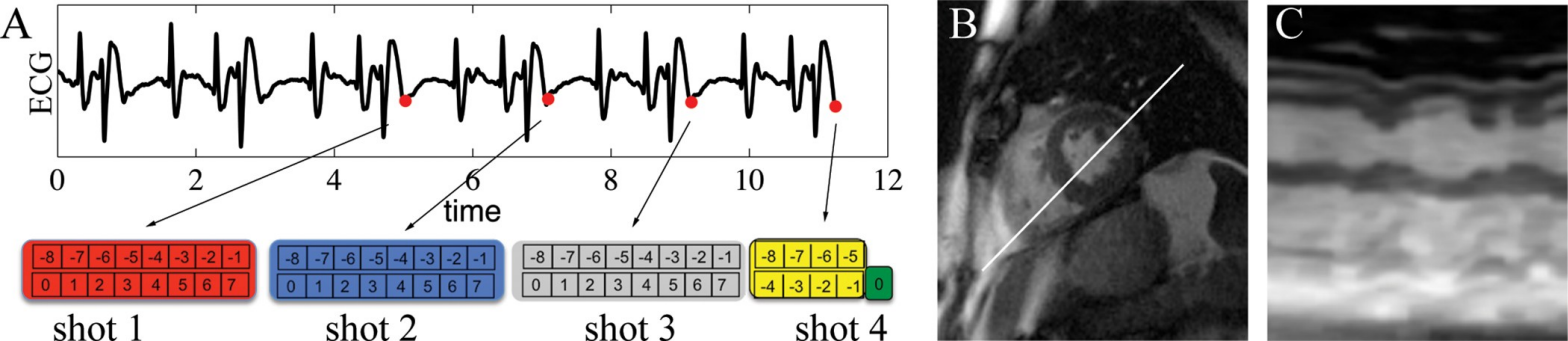
Utility of the proposed approach when imaging a patient with arrhythmia. **A)** The cross-correlation based approach robustly identifies similar periods in the cardiac cycle in the setting of arrhythmia. **B)** A high-quality short axis image can be generated with the closed-loop scheme. **C)** The temporal projection illustrates the effect of complex rhythm on wall motion. This suggests the close-loop approach could enable multi-shot imaging of patients with complex rhythms.

To demonstrate the potential utility of this approach in imaging the heart of patients who have an arrhythmia, we utilized previously collected ECG and golden angle radial data (**Fig 4**) to demonstrate the closed-loop approach in the setting of a complex ECG. The cross-correlation algorithm (**Fig 4A**) correctly identified the correct phase in the cardiac cycle despite the complex arrhythmia seen on the ECG. While the data was acquired with golden-angle views, we calculated the angles the closed-loop approach would prescribe and show the short-axis (**Fig 4B**) and temporal projection images (**Fig 4C**). This suggests our approach could be used to increase the image quality of real-time images in patients with complex rhythms.

## Discussion

We developed and investigated an autonomous k-space trajectory control system for cardiac MRI that implements closed-loop feedback. There were several important conceptual and technical aspects to this investigation. To our knowledge, this was the first implementation of an autonomous k-space trajectory using ECG and k-space trajectory feedback integrated in a closed-loop for segmented cine MRI. The algorithm was successfully implemented on a clinical whole-body MRI scanner as embedded and real-time software and its feasibility was shown in segmented cardiac cine MRI in healthy normal subjects. Our results showed that it enabled real-time cardiac MRI with good spatial and temporal resolution and reduced radial undersampling artifacts compared to conventional open-loop radial acquisitions.

The closed-loop radial trajectory is different from other radial sampling trajectories in that the view angle are not predetermined. For example, in an acquisition with fixed angular spacing Δ*θ*, the view angles increase from 0° to 180°- Δ*θ*. In a segmented acquisition with ten segments per heartbeat, the angles 0, Δ*θ*, 2Δ*θ*, …, 10Δ*θ* will be repeated for each frame of the first heartbeat and 11Δ*θ*, 12Δ*θ*, …, 20Δ*θ* for the second heartbeat, and so on, until all radial views are acquired. The total number of radial views *N* = 180/Δ*θ*, where both the angular spacing Δ*θ* and number of view angles *N* is determined before the scan begins. While this approach leads to the optimal sampling of k-space, the performance is substantially degraded if 1) one or more segments are not acquired due to arrhythmia rejection or 2) if significant motion–such as breathing–occurs due to arrythmia rejection causing a prolonged acquisition.

Similarly, in a golden angle trajectory [13], there is a fixed angular spacing Δ*θ* = 111.25° and each new angle is set by a schedule 0, Δ*θ*, 2Δ*θ*, …, and so on, before the scan begins. In the closed-loop trajectory, these view angles were not known in advance, but calculated on-the-fly using previous physiologic and k-space data. A limitation of predefined trajectories was that real-time physiologic information from the patient or knowledge about what and when data was collected was not used to judiciously inform the acquisition of new data. As was shown in the results, this led to sub-optimal k-space sampling behavior. In particular, golden radial sampling trajectory showed degraded image quality due to sub-optimal uniformity. Of particular note is the high variability in sampling across multiple cardiac periods (Table 2). The closed-loop sampling overcomes these limitations and provides improved uniformity and low variability in k-space sampling, permitting the synthesis of data across multiple cardiac periods for real-time acquisition and display with high image quality and low temporal footprint.

We observed some important differences between conventional segmented k-space sampling strategies and one that used a closed-loop. In segmented cine MRI, k-space data across several heartbeats would be combined to make a single dataset showing a single heartbeat. However, our approach has elements of both segmented and real-time acquisitions. Each image represented data from the previous 4 heartbeats, similar to how cine MRI would combine data from heartbeats, but the autonomous scan also results in images from every heart beat such as in a real-time acquisition. Furthermore, segmented cine MRI does not include closed-loop feedback systems for cardiac physiologic feedback such as from the ECG or from cardiac navigator signals. While prospective cine MRI certainly uses ECG feedback to provide synchronization for gating, it is open-loop since Cartesian spin warp imaging marches through a predefined list of phase encoding gradients [4, 6] and non-Cartesian spatial encoding is performed using a similarly predefined list of view angles or spirals. In retrospectively gated reconstructions [6], ECG and navigator signals are used to properly bin acquired data into the correct cardiac or respirator phase, but they do not direct the sequence to update its trajectory in response to new information. Furthermore, both prospective and retrospectively gated cine MRI do not measure the output sampling trajectory nor do they maintain a desired setpoint for maintaining uniformity of k-space sampling across multiple heartbeats.

In real-time, interactive MRI, data is sampled as quickly as possible and images are displayed as soon as sufficient data has been collected. However, in many clinical applications, it is challenging to sample real-time MRI data with sufficient signal-to-noise ratio and spatial resolution without compromising temporal resolution. Furthermore, while recent advances in parallel and sparse scan acceleration techniques enable the collection of 2D images in real-time with good spatial and temporal resolution [1, 28], image fidelity is degraded in real-time 3D applications. This framework could be adapted to improve both of these clinical applications.

The ECG is routinely acquired in 1.5T and 3T scanners for cardiac gating and the signals we used were recorded in a 1.5T scanner. The cross-correlation approach robustly identified the correct phase of the cardiac cycle despite the known magnetohemodynamic effect on the signal. Given the pattern-matching nature of the cross-correlation, it seems likely that the approach could work in the setting of more significant distortions (for example, at 7T) as well as with other signals such as acoustic waveforms from a stethoscope. Future work could assess the cross-correlation gating as well as the closed loop imaging at higher fields and with other signals.

This approach builds on features of other MRI techniques such automatic scanning [29, 30] and view planning [29, 31–33], inadvertent patient motion correction [5], and respiratory motion informed k-space sampling [34, 35]. Furthermore, some acquisitions use internal sensors to measure physiologic motion from k-space, image space navigators [5, 36–39] or self-navigation [40] while other techniques use external sensors to capture motion information such as radiofrequency coils, ultrasound devices [41] and optical tracking devices [42, 43]. Future work could integrate these physiologic signals into the closed-loop approach we describe.

While our results demonstrated the feasibility of an adaptive real-time system for cardiovascular MRI, additional work is needed to bring this technology into clinical use. The TR achieved with our approach was long compared to the clinical standard values. Given the fast calculation time of the algorithm, this is primarily due to implementation of the approach on various computer systems with multiple network interfaces. Optimizing these interfaces or implementing the algorithm directly on the scanner hardware is expected to enable faster TRs. It is also expected that faster TRs would enable improved uniformity in sampling due to increase sampling density. Data should be gathered from patients to confirm that the real-time system provides accurate and reproducible beat-to-beat assessment of left ventricular function. Comparison of the sequence in patients with reduced ejection fraction or with left ventricular dyssynchrony should be performed to verify that temporal fidelity is sufficient to characterize compromised function or ventricular wall motion abnormalities. Further optimization of radiofrequency and gradient pulse durations is necessary to further reduce the sequence temporal footprint. The system should be integrated with a balanced steady-state free-precession pulse sequence because of its superior contrast, signal-to-noise ratio, and temporal resolution compared to spoiled gradient echo pulse sequences at 1.5 T. The cross-correlation algorithm appears to work well for subjects in sinus rhythms, however the length of the historical ECG data should be optimized for patients with arrhythmias or for 3D and interventional applications.

We implemented our technique in the setting of 2D radial k-space sampling for cine imaging since there is a straightforward definition of the optimal angle for subsequent acquisition. However, Cartesian and 3D sampling trajectories could both benefit from this approach. Furthermore, parametric mapping acquisitions such as T1-, T2-mapping and perfusion imaging are applications we plan to explore in future work. Lastly, this approach optimized sampling given past data acquisition without the expectation of future data acquisition. Other applications may allow for this assumption to be relaxed by modeling future data acquisition and further improve performance.

## Conclusions

We present an initial implementation of a closed-loop controller that defines radial k-space sampling. Based on recordings of ECGs in the MRI as well as 4 in vivo scans, the approach enables segmented acquisitions with improved sampling uniformity relative to the retrospective sorting of golden angle data. Furthermore, initial findings for a patient with arrhythmias suggest the approach would enable scanning of complex rhythms.

## Supporting information

S1 Fig Closed-loop identification of prior cardiac phases. **A**) (top) A brief portion of ECG signal is compared to the entire scan ECG signal (bottom) via cross correlation. **B**) (top) Cross-correlation output. Local peaks are indicated in red. (bottom) In this segmented acquisition, MRI data from 4 beats was used for reconstruction. Radial views from shots 1 through 3 (red, blue and gray) as well as the most recent views (yellow) are used to determine the newest segment (green). The number of radial views obtained from each shot (e.g. 8 views/shot) can be varied depending on the application.
(TIF)

S2 Fig Overview of stages of approach. Training mode A consists of TCP/IP reading of new ECG data, buffer storage of ECG sampled, and other software overhead. Training mode B occurs once the buffers are populated and includes calculation of the cross correlation to identify similar periods of ECG signal. During training mode B, the optimal angle is not calculated since insufficient beats are identified. Active mode occurs after both the buffers are populated and sufficient number of beats can be identified. It includes calculation of the optimal sampling angle.
(TIF)

S3 Fig Radial k-space sampling uniformity analysis. **A**, histogram of adjacent view angle difference Δ*θ* for uniform (black) and random (red) radial sampling trajectories. The uniform sampling distribution is a delta function positioned at the *π*/*N*_*θ*_. **B**, cumulative sum (CS) of uniform (black) and random (red) radial sampling trajectories. The uniformity metric *U* is the ratio of the shaded areas.
(TIF)

S1 Movie ECG sampling, results of the cross-correlation, k-space sampling, and imaging results over 10 seconds for the patient shown in Fig 2. The ECG vs time is shown on the top row with the overlay of beats identified using the cross-correlation shown in the right hang corner. The distribution of angles is shown in the bottom left and demonstrates and increase in uniformity as the scheme moves from golden-angle to ARKS sampling. Reconstructed images are shown in the bottom right which show cardiac motion and an improvement with improved sampling uniformity.
(M4V)

S2 Movie Comparison of single-shot and ARKS multi-shot imaging. The autonomous trajectory has both elements of a segmented scan and a real-time scan. The first and second columns show single-short approaches with a small temporal footprint (37.2 ms) and long temporal footprint (285 ms), respectively. As was expected, the image with 6 views showed low contrast and high noise while the high temporal footprint (46 view) image blurred cardiac motion (especially as depicted by the projection through the ventricle). The autonomous segmented scan acquired 46 views across 8 heartbeats (6 views per heartbeat) and showed a high-quality segmented scan with high temporal fidelity and real-time display.
(M4V)

## List of abbreviations

DSP: digital signal process
ECG: electrocardiogram
FOV: field-of-view
RF: radiofrequency
PSF: point spread function
TE: echo time
TR: repetition time

## References

[1] Zhang S, Block KT, Frahm J. Magnetic resonance imaging in real time: advances using radial FLASH. J Magn Reson Imaging. 2010;31: 101–9. 10.1002/jmri.21987 19938046

[2] Narayanan S, Nayak K, Lee S, Sethy A, Byrd D. An approach to real-time magnetic resonance imaging for speech production. J Acoust Soc Am. 2004;115: 1771–1776. 10.1121/1.1652588 15101655

[3] Hundley WG, Bluemke DA, Finn JP, Flamm SD, Fogel M a, Friedrich MG, et al ACCF/ACR/AHA/NASCI/SCMR 2010 expert consensus document on cardiovascular magnetic resonance: a report of the American College of Cardiology Foundation Task Force on Expert Consensus Documents. Circulation. 2010;121: 2462–508. 10.1161/CIR.0b013e3181d44a8f 20479157 PMC3034132

[4] Glover GH, Pelc NJ. A rapid-gated cine MRI technique. Magn Reson Annu. 1988; 299–333. Available: http://www.ncbi.nlm.nih.gov/pubmed/3079300 3079300

[5] Ehman RL, Felmlee JP. Adaptive technique for high-definition MR imaging of moving structures. Radiology. 1989;173: 255–263. 10.1148/radiology.173.1.2781017 2781017

[6] Lenz GW, Haacke EM, White R. Retrospective Cardiac Gating: A Review of Technical Apsects and Future Directions. Magn Reson Imaging. 1989;I: 445–455. Available: http://www.sciencedirect.com/science/article/pii/0730725X89903998 10.1016/0730-725x(89)90399-8 2607896

[7] Lauterbur PC. Image formation by induced local interactions. Examples employing nuclear magnetic resonance. 1973. Clin Orthop Relat Res. 1989; 3–6. 2663289

[8] Bergin CJ, Glover GH, Pauly JM. Lung parenchyma: magnetic susceptibility in MR imaging. Radiology. 1991;180: 845–848. 10.1148/radiology.180.3.1871305 1871305

[9] Glover GH, Pauly JM. Projection Reconstruction Techniques for Reduction of Motion Effects in MRI. Magn Reson Med. 1992;28: 275–289. 10.1002/mrm.1910280209 1461126

[10] Seiberlich N, Breuer FA, Blaimer M, Barkauskas K, Jakob PM, Griswold MA. Non-Cartesian data reconstruction using GRAPPA operator gridding (GROG). Magn Reson Med. 2007;58: 1257–1265. 10.1002/mrm.21435 17969027

[11] Ahn CB, Kim JH, Cho ZH. High-speed spiral-scan echo planar NMR imaging-I. IEEE Trans Med Imaging. 1986;5: 2–7. 10.1109/TMI.1986.4307732 18243976

[12] Meyer CH, Hu BS, Nishimura DG, Macovski A. Fast spiral coronary artery imaging. Magn Reson Med. 1992;28: 202–213. 10.1002/mrm.1910280204 1461123

[13] Winkelmann S, Schaeffter T, Koehler T, Eggers H, Doessel O. An optimal radial profile order based on the Golden Ratio for time-resolved MRI. IEEE Trans Med Imaging. 2007;26: 68–76. 10.1109/TMI.2006.885337 17243585

[14] Contijoch F, Iyer SK, Pilla JJ, Yushkevich P, Gorman JH, Gorman RC, et al Self-gated MRI of multiple beat morphologies in the presence of arrhythmias. Magn Reson Med. 2016; n/a—n/a. 10.1002/mrm.26381 27579717 PMC5332534

[15] Contijoch F, Witschey WRT, Rogers K, Rears H, Hansen M, Yushkevich P, et al User-initialized active contour segmentation and golden-angle real-time cardiovascular magnetic resonance enable accurate assessment of LV function in patients with sinus rhythm and arrhythmias. J Cardiovasc Magn Reson. 2015;17: 37 10.1186/s12968-015-0146-9 25994390 PMC4440288

[16] Han F, Zhou Z, Rapacchi S, Nguyen K-L, Finn JP, Hu P. Segmented golden ratio radial reordering with variable temporal resolution for dynamic cardiac MRI. Magn Reson Med. 2016;76: 94–103. 10.1002/mrm.25861 26243442 PMC4740269

[17] Chava R, Assis F, Herzka D, Kolandaivelu A. Segmented radial cardiac MRI during arrhythmia using retrospective electrocardiogram and respiratory gating. Magn Reson Med. 2018; 1–13. 10.1002/mrm.27533 30362588

[18] Contijoch F, Han Y, Hansen M, Kellman P, Gualtieri E, Elliott M, et al Continuous adaptive radial sampling of k-space from real-time physiologic feedback in MRI. J Cardiovasc Magn Reson. 2015;17: P37 10.1186/1532-429X-17-S1-P37

[19] Walsh DO, Gmitro AF, Marcellin MW. Adaptive reconstruction of phased array MR imagery. Magn Reson Med. 2000;43: 682–90. 10.1002/(sici)1522-2594(200005)43:5&lt;682::aid-mrm10&gt;3.0.co;2-g 10800033

[20] Pruessmann K, Weiger M. Advances in sensitivity encoding with arbitrary k‐space trajectories. Magn Reson …. 2001;651: 638–651. 10.1002/mrm.1241 11590639

[21] Hansen MS, Sorensen TS. Gadgetron: an open source framework for medical image reconstruction. Magn Reson Med. 2013;69: 1768–1776. 10.1002/mrm.24389 22791598

[22] Contijoch F, Iyer SK, Pilla JJ, Yushkevich P, Gorman JH, Gorman RC, et al Self-gated MRI of multiple beat morphologies in the presence of arrhythmias. Magn Reson Med. 2017;78: 678–688. 10.1002/mrm.26381 27579717 PMC5332534

[23] Beck A, Teboulle M. A Fast Iterative Shrinkage-Thresholding Algorithm for Linear Inverse Problems. SIAM J Imaging Sci. 2009;2: 183–202. 10.1137/080716542

[24] Kamesh Iyer S, Tasdizen T, Likhite D, DiBella E. Split Bregman multicoil accelerated reconstruction technique: A new framework for rapid reconstruction of cardiac perfusion MRI. Med Phys. 2016;43: 1969–1981. 10.1118/1.4943643 27036592 PMC4818275

[25] Lauzon ML, Lauzon ML, Rutt BK, Rutt BK. Effects of polar sampling in k-space. Magn Reson Med. 1996;36: 940–9. 10.1002/mrm.1910360617 8946360

[26] Peters D, Rohatgi P. Characterizing Radial Undersampling Artifacts for Cardiac Applications. Magn Reson Med. 2006;403: 396–403. 10.1002/mrm.20782 16408266

[27] Scheffler K, Hennig J. Reduced circular field-of-view imaging. Magn Reson Med. 1998;40: 474–480. 10.1002/mrm.1910400319 9727952

[28] Witschey WRT, Contijoch F, McGarvey JR, Ferrari VA, Hansen MS, Lee ME, et al Real-Time Magnetic Resonance Imaging Technique for Determining Left Ventricle Pressure-Volume Loops. Ann Thorac Surg. 2014;97: 1597–1603. 10.1016/j.athoracsur.2014.01.010 24629301 PMC3939798

[29] Lu X, Jolly MP, Georgescu B, Hayes C, Speier P, Schmidt M, et al Automatic view planning for cardiac MRI acquisition. Lect Notes Comput Sci (including Subser Lect Notes Artif Intell Lect Notes Bioinformatics). 2011;6893 LNCS: 479–486. 10.1007/978-3-642-23626-6_59 22003734

[30] Moenninghoff C, Umutlu L, Kloeters C, Ringelstein A, Ladd ME, Sombetzki A, et al Workflow efficiency of two 1.5 T MR scanners with and without an automated user interface for head examinations. Acad Radiol. 2013;20: 721–730. 10.1016/j.acra.2013.01.004 23473722

[31] Nitta S, Takeguchi T, Matsumoto N, Kuhara S, Yokoyama K, Ishimura R, et al Automatic slice alignment method for cardiac magnetic resonance imaging. MAGMA. 2013;26: 451–461. 10.1007/s10334-012-0361-4 23354512

[32] Lelieveldt BP, van der Geest RJ, Lamb HJ, Kayser HW, Reiber JH. Automated observer-independent acquisition of cardiac short-axis MR images: a pilot study. Radiology. 2001;221: 537–542. 10.1148/radiol.2212010177 11687701

[33] Jackson C, Robson M, Francis J, Noble JA. Automatic Planning of the Acquisition of Cardiac MR Images. Computerized Medical Imaging and Graphics. 2003 pp. 541–548. 10.1007/978-3-540-39899-8_67 15464880

[34] Bailes DR, Gilderdale DJ, Bydder GM, Collins a G, Firmin DN. Respiratory ordered phase encoding (ROPE): a method for reducing respiratory motion artefacts in MR imaging. Journal of computer assisted tomography. 1985 pp. 835–838. 10.1097/00004728-198507010-00039 4019854

[35] Markl M, Harloff A, Bley TA, Zaitsev M, Jung B, Weigang E, et al Time-resolved 3D MR velocity mapping at 3T: improved navigator-gated assessment of vascular anatomy and blood flow. J Magn Reson Imaging. 2007;25: 824–831. 10.1002/jmri.20871 17345635

[36] Welch EB, Manduca A, Grimm RC, Ward HA, Jack CRJ. Spherical navigator echoes for full 3D rigid body motion measurement in MRI. Magn Reson Med. 2002;47: 32–41. 10.1002/mrm.10012 11754440

[37] Norris DG, Driesel W. Online motion correction for diffusion-weighted imaging using navigator echoes: Application to RARE imaging without sensitivity loss. Magn Reson Med. 2001;45: 729–733. 10.1002/mrm.1099 11323797

[38] Korin HW, Felmlee JP, Ehman RL, Riederer SJ. Adaptive technique for three-dimensional MR imaging of moving structures. Radiology. 1990;177: 217–221. 10.1148/radiology.177.1.2399320 2399320

[39] Fu ZW, Wang Y, Grimm RC, Rossman PJ, Felmlee JP, Riederer SJ, et al Orbital navigator echoes for motion measurements in magnetic resonance imaging. Magn Reson Med. 1995;34: 746–753. 10.1002/mrm.1910340514 8544696

[40] Uribe S, Muthurangu V, Boubertakh R, Schaeffter T, Razavi R, Hill DLG, et al Whole-heart cine MRI using real-time respiratory self-gating. Magn Reson Med. 2007;57: 606–13. 10.1002/mrm.21156 17326164

[41] Feinberg DA, Giese D, Bongers DA, Ramanna S, Zaitsev M, Markl M, et al Hybrid ultrasound MRI for improved cardiac imaging and real-time respiration control. Magn Reson Med. 2010;63: 290–296. 10.1002/mrm.22250 20025068 PMC2813925

[42] Schulz J, Siegert T, Reimer E, Labadie C, Maclaren J, Herbst M, et al An embedded optical tracking system for motion-corrected magnetic resonance imaging at 7T. MAGMA. 2012;25: 443–453. 10.1007/s10334-012-0320-0 22695771

[43] Ooi MB, Krueger S, Thomas WJ, Swaminathan S V, Brown TR. Prospective real-time correction for arbitrary head motion using active markers. Magn Reson Med. 2009;62: 943–954. 10.1002/mrm.22082 19488989 PMC3033410

