# Supplementary figures and images for: Closed-loop control of k-space sampling via physiologic feedback for cine MRI
Source: PLoS One. 2020 Dec 29;15(12):e0244286. doi: 10.1371/journal.pone.0244286 (PMC7771662; doi:10.1371/journal.pone.0244286)

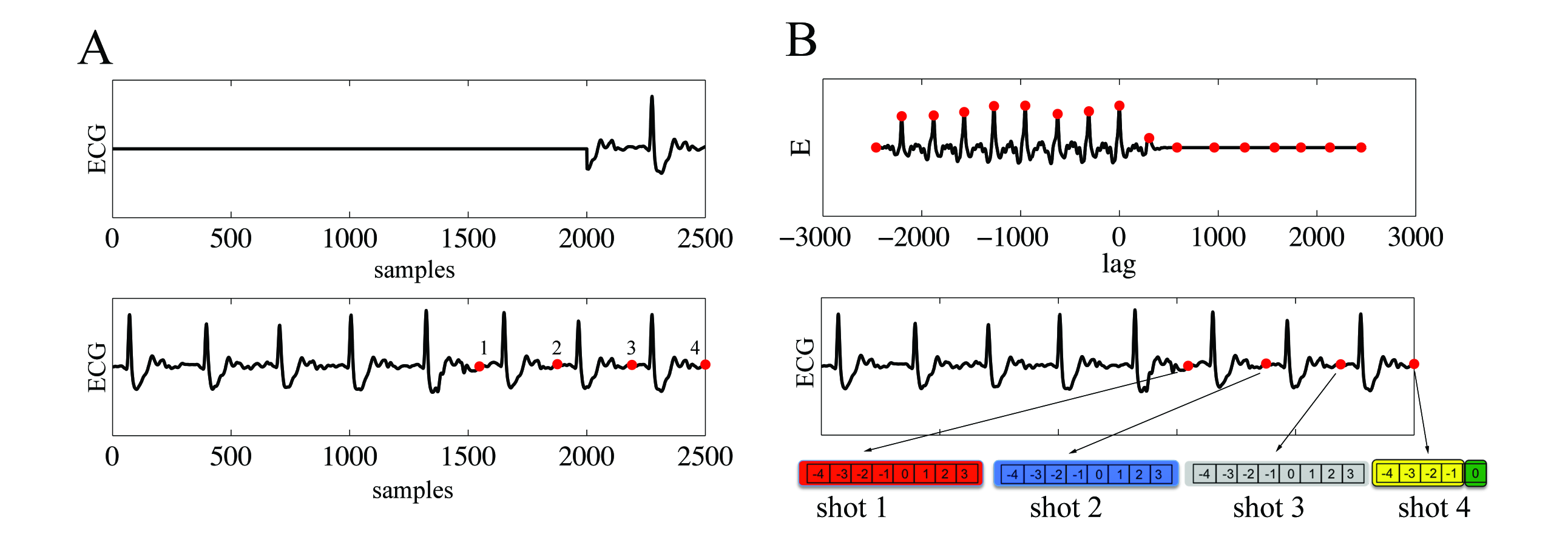

Supplement: S1 Fig — A) (top) A brief portion of ECG signal is compared to the entire scan ECG signal (bottom) via cross correlation. B) (top) Cross-correlation output. Local peaks are indicated in red. (bottom) In this segmented acquisition, MRI data from 4 beats was used for reconstruction. Radial views from shots 1 through 3 (red, blue and gray) as well as the most recent views (yellow) are used to determine the newest segment (green). The number of radial views obtained from each shot (e.g. 8 views/shot) can be varied depending on the application. (TIF) [file pone.0244286.s001.tif]

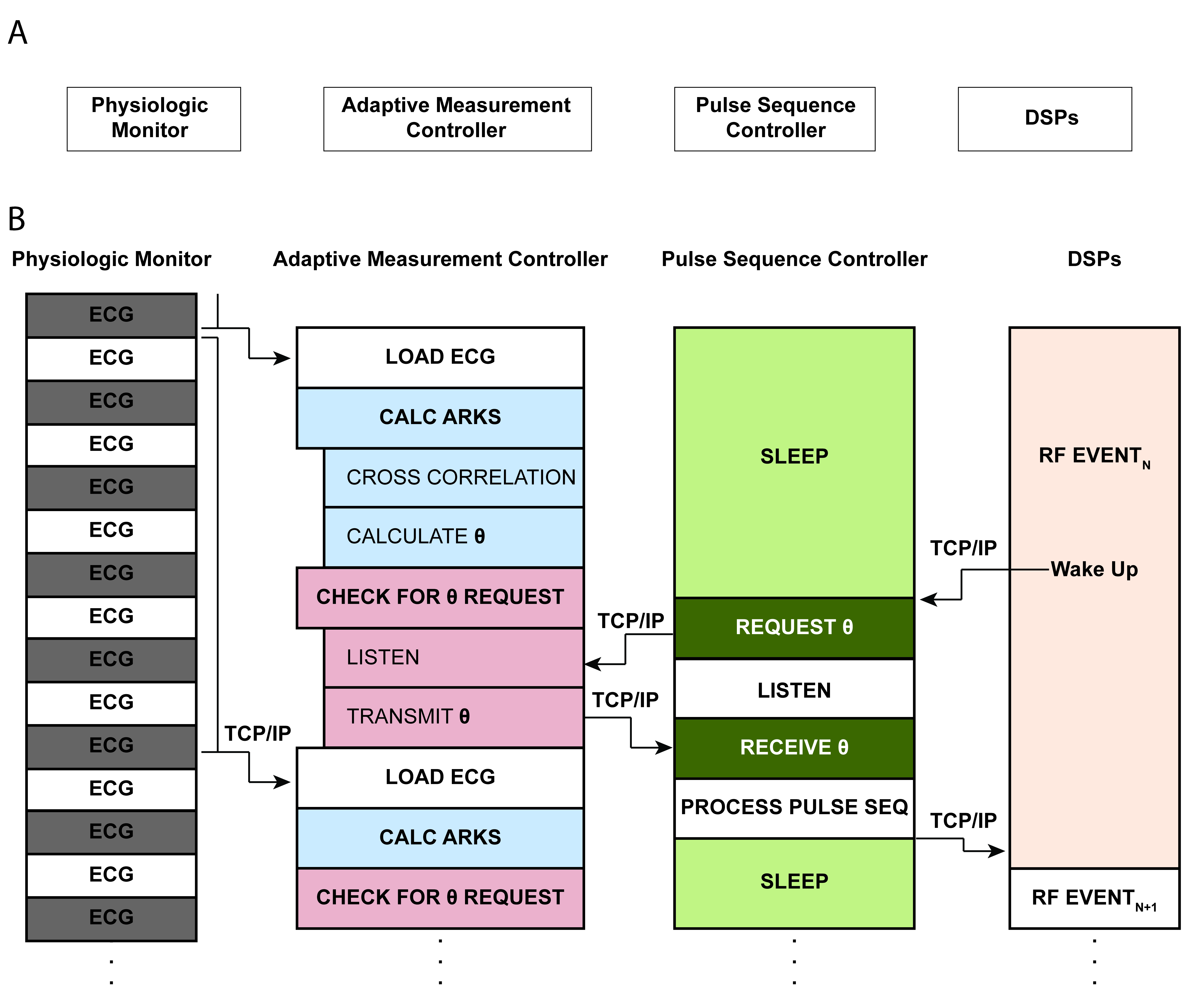

Supplement: S2 Fig — Training mode A consists of TCP/IP reading of new ECG data, buffer storage of ECG sampled, and other software overhead. Training mode B occurs once the buffers are populated and includes calculation of the cross correlation to identify similar periods of ECG signal. During training mode B, the optimal angle is not calculated since insufficient beats are identified. Active mode occurs after both the buffers are populated and sufficient number of beats can be identified. It includes calculation of the optimal sampling angle. (TIF) [file pone.0244286.s002.tif]

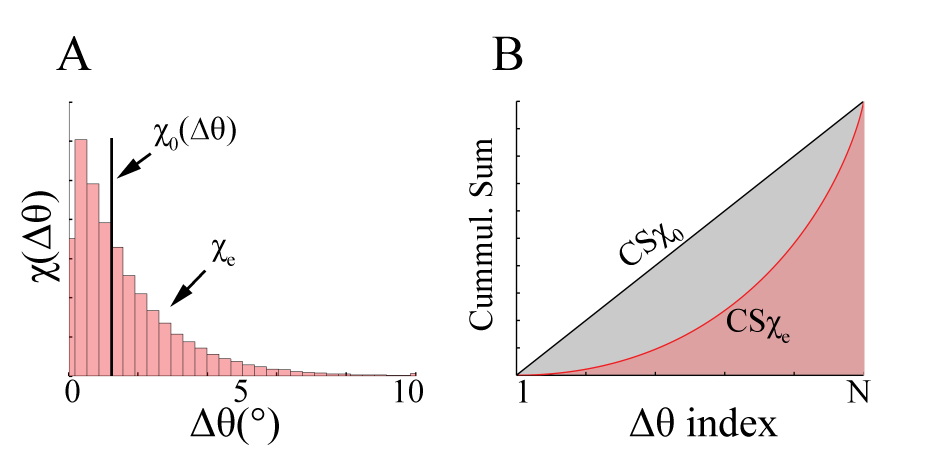

Supplement: S3 Fig — A, histogram of adjacent view angle difference Δθ for uniform (black) and random (red) radial sampling trajectories. The uniform sampling distribution is a delta function positioned at the π/Nθ. B, cumulative sum (CS) of uniform (black) and random (red) radial sampling trajectories. The uniformity metric U is the ratio of the shaded areas. (TIF) [file pone.0244286.s003.tif]
